# The establishment of ecological conservation for herpetofauna species in hotspot areas of South Korea

**DOI:** 10.1038/s41598-022-19129-0

**Published:** 2022-09-01

**Authors:** Min Seock Do, Seok-Jun Son, Green Choi, Nakyung Yoo, Dae-in Kim, Kyo-Soung Koo, Hyung-Kyu Nam

**Affiliations:** 1 National Institute of Biological Resources, Seo-gu, Incheon, 22689 South Korea; 2 Korea Institute of Wildlife Ecology, Daejeon, 34388 Republic of Korea; 3 GREEN Together Institute, Seocheon, 33646 Republic of Korea; 4 National Institute of Ecology, Yeongyang, 36541 Republic of Korea; 5 HERPING, Seoul, 02505 Republic of Korea; 6 Interdisciplinary Program of EcoCreative, Ewha Woman’s University, Seoul, 07804 Republic of Korea

**Keywords:** Ecology, Conservation biology, Ecological modelling, Forestry

## Abstract

Understanding the geographic distribution of species is crucial for establishing protected areas. This study aimed to identify the preferred habitat environment of South Korean herpetofauna using distribution point information, providing the information necessary to protect their habitat by establishing a species distribution model. We found that climate variables in the region where 19 amphibians and 20 reptiles were distributed correlated with the altitude, suggesting that altitude had a major influence on their distribution. The species distribution modeling indicated that 10–12 amphibian and 13–16 reptile species inhabit the Gangwon-do region, forming hotspot areas in the eastern and western regions around the Taebaek Mountains. Some of these hotspot areas occurred in the Demilitarized Zone and national parks, which are government-managed ecological conservation areas. However, some hotspot areas are vulnerable to habitat destruction due to development and deforestation as they are not designated conservation areas. Therefore, it is necessary to establish new conservation areas with a focus on herpetofauna after confirming the actual inhabitation of species through precise monitoring in predicted hotspot areas and designating them as protected areas. Our results can serve as important basic data for establishing protection measures and designating protected areas for herpetofauna species.

## Introduction

Understanding the geographic distribution of species is becoming an important factor not just in academic domains such as evolutionary and conservation biology but also in numerous applied sectors, such as the establishment of protected areas and management of invasive species^1–3^. While many countries are making efforts to investigate species distribution, it is almost impossible to obtain precise species distribution data for a wide area at the national level because of human-related constraints as well as technical, temporal, and financial limitations^4–7^. Species distribution modeling (SDM), used to predict species distribution based on observation and local environmental data, can compensate for the limitations of observation data and has recently been used in various studies^8–10^.

Because of their role as prey for birds, fish, and mammals, as well as their role as predators of terrestrial and aquatic insects, herpetofauna species play an important role in the conservation of biodiversity in the intermediate position of the ecosystem food chain^11–14^. Furthermore, terrestrial herpetofauna species are known to be vulnerable to habitat destruction and climate change because of their short migration distance and limited dispersal ability, which are consequences of their narrow range of motion compared to other vertebrates^15–17^. Therefore, understanding the geographical distribution of herpetofauna species is essential for their conservation, and various modeling techniques have been applied and evaluated to determine their distribution characteristics^4,18,19^. These studies have mainly focused on establishing strategies for species conservation and protection by predicting habitat changes as a consequence of climate change or identifying hotspots or core areas^2,4,6,20^.

To date, 20 species of amphibians belonging to two orders and seven families and 31 species of reptiles belonging to two orders and 11 families have been reported from South Korea. Among them, 20 species live on land, excluding those inhabiting the marine area^21^. In the Korean Red List of Threatened Species, a total of 10 species, including five amphibians and five reptiles, requiring protection because of habitat destruction and population decline related to industrial development are designated as Endangered (EN) and Vulnerable (VU). The Ministry of Environment of the Republic of Korea has also designated and protected seven herpetofauna species inhabiting South Korea as *endangered wild species*, with two class I species at a high risk of extinction and five class II species at possible risk of extinction^22,23^.

In South Korea, various studies have investigated the geographical distribution patterns and habitat characteristics of herpetofauna species^24–27^. Recently, studies on habitat prediction and climate change using species distribution models have also been conducted^28–34^. The hotspot areas for herpetofauna species in South Korea were identified to be paddy wetlands around the coastal areas of Gyeonggi-do and Chungcheongnam-do, which are located in the western region of the Korean Peninsula, and they are used as important basic data for establishing protection measures when designating protected areas^7,27^. Nevertheless, most studies on the distribution characteristics of herpetofauna species conducted to date have focused on a single species or genus, while areas with a high diversity of all taxa have rarely been investigated and protected^7,32–34^.

The present study aimed to (1) identify the distribution of herpetofauna species using observation data from South Korea and species distribution modeling and (2) provide information necessary to protect their habitats by determining habitat requirements for each species; this was done by extracting environmental variables such as altitude and climate of their distribution points as well as correlations between these variables. Subsequently, to compensate for the limitations of the observation data, we (3) created a species distribution model using the Maxent model to predict suitable habitats, and (4) identified the regions most diversely inhabited by herpetofauna species by superimposing the models as administrative units (provinces) to facilitate species conservation and management. Finally, we established a detailed management plan by comparing the obtained results with the current status of herpetofauna species protection in South Korea.

## Results

### Habitat distribution characteristics

The 19 amphibian and 20 reptile species inhabiting South Korea were observed at 25,400 and 8,581 locations, respectively (Table 1). *Rana nigromaculata* was the most commonly identified (5306 locations), and *Hynobius yangi* was the least commonly identified (21 locations) amphibian species, and *Rhabdophis tigrina* was the most commonly identified (2121 locations), and *Eremias argus* was the least commonly identified (212 locations) reptile species (Table 1).

**Table 1:** The 19 amphibian species and 20 reptile species included in this study, along with the number of collection sites used for Maxent modeling.

Order	Family	Species	State			Number of location source				Note	Total sites
			KRL^a^	KMOE^b^	IUCN^c^	NES^d^	NRS^e^	GBIF^f^	Study source (site)		
**Amphibia**											
**Caudata**											
	Hynobiidae	*Hynobius leechii*	LC	–	LC	2658				–	2658
	Hynobiidae	*Hynobius yangi*	EN	II	EN		3	18		Endemic	21
	Hynobiidae	*Hynobius quelpaertensis*	NT	–	VU	125		49		Endemic	174
	Hynobiidae	*Hynobius unisacculus*	VU	–	EN			50		Endemic	50
	Hynobiidae	*Onychodactylus koreanus*	LC	–	–	198				Endemic	198
	Plethodontidae	*Karsenia koreana*	NT	–	LC	10	3	92		Endemic	105
**Anura**											
	Discoglossidae	*Bombina orientalis*	LC	–	LC	2771				–	2771
	Bufonidae	*Bufo gargarizans*	LC	–	LC	1116				–	1116
	Bufonidae	*Bufo stejnegeri*	LC	–	LC	211				–	211
	Hylidae	*Dryophytes japonica*	LC	–	LC	4070				–	4070
	Hylidae	*Dryophytes suweonensis*	EN	I	EN	1		77		Endemic	78
	Microhylidae	*Kaloula borealis*	VU	II	LC	80	1	31		–	112
	Ranidae	*Pelophylax nigromaculatus*	LC	–	NT	5306				–	5306
	Ranidae	*Pelophylax chosenicus*	VU	II	VU	20		47		Endemic	67
	Ranidae	*Rana coreana*	LC	–	LC	1332				–	1332
	Ranidae	*Rana uenoi*	LC	–	LC	3007				–	3007
	Ranidae	*Rana huanrenensis*	LC	–	LC	657				–	657
	Ranidae	*Glandirana rugosa*	LC	–	LC	1397				–	1397
	Ranidae	*Lithobates catesbeianus*	–	–	LC	2070				Invasive	2070
**Reptilia**											
**Testudinata**											
	Trionychidae	*Pelodiscus maackii*	VU	–	–	60				–	60
	Emydidae	*Mauremys reevesii*	VU	II	EN	28				–	28
	Emydidae	*Trachemys scripta elegans*	–	–	–	107				Invasive	107
**Squamata (Lacertilia)**											
	Gekkonidae	*Gekko japonicus*	NA	–	LC				^38^ (244)	–	244
	Scincidae	*Scincella vandenburghi*	LC	–	LC	297				–	297
	Scincidae	*Scincella huanrenensis*	NT	–	CR	30				–	30
	Lacertidae	*Takydromus amurensis*	LC	–	–	673				–	673
	Lacertidae	*Takydromus wolteri*	LC	–	–	660				–	660
	Lacertidae	*Eremias argus*	VU	II	–	16		6		–	22
**Squamata (Serpentes)**											
	Colubridae	*Oocatochus rufodorsatus*	LC	–	LC	521				–	521
	Colubridae	*Elaphe dione*	LC	–	LC	1112				–	1112
	Colubridae	*Elaphe schrenckii*	VU	II	–	70	6	19		–	95
	Colubridae	*Rhabdophis tigrinus*	LC	–	–	2121				–	2121
	Colubridae	*Hebius vibakari*	LC	–	–	125				–	125
	Colubridae	*Sibynophis chinensis*	EN	I	LC				^32^ (33)	–	33
	Colubridae	*Lycodon rufozonatus*	LC	–	LC	479				–	479
	Colubridae	*Orientocoluber spinalis*	NT	–	–	51				–	51
	Viperidae	*Gloydius ussuriensis*	LC	–	–	1194				–	1194
	Viperidae	*Gloydius brevicaudus*	LC	–	–	629				–	629
	Viperidae	*Gloydius intermedius*	LC	–	LC	100				–	100

^a^Status according to the Koran Red List of Threatened Species (KRL), ^b^Status of Endangered Species designated by the KMOE (Korea Ministry of Environment). ^c^Location source of Nationwide Environmental Study (NES), ^d^Location source of Natural Resource Study (NRS), ^e^Location source of Global Biodiversity Information Facility (GBIF).

**Figure 1:**
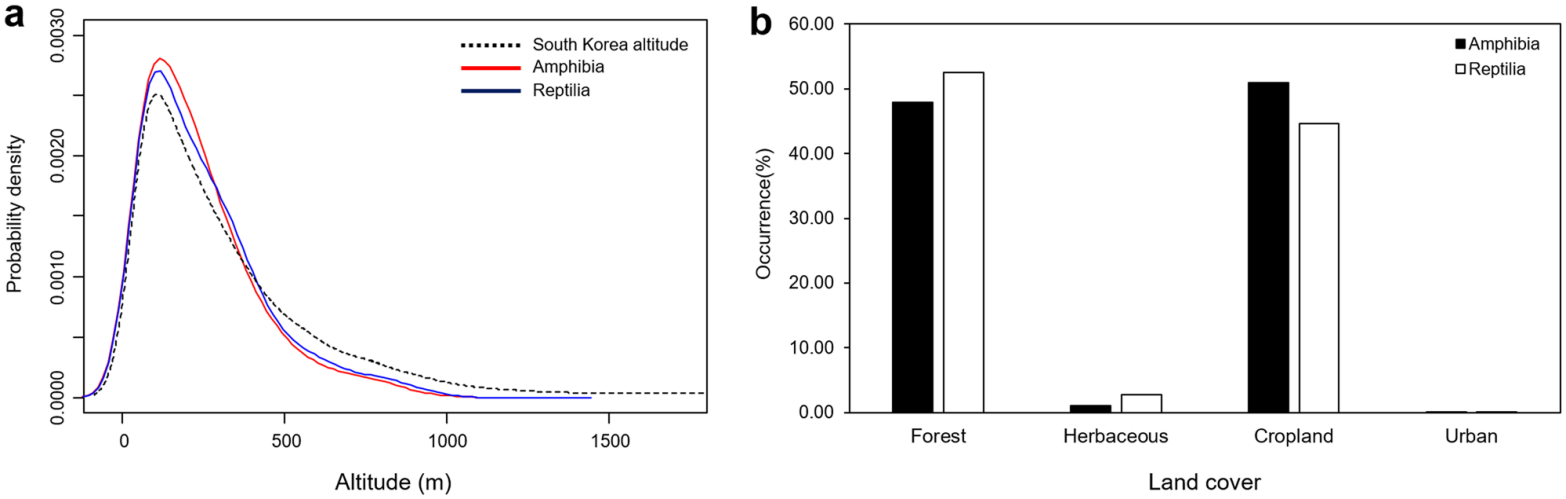
(**a**) Kernel density plot of the occurrence of the 19 amphibian and 20 reptile species according to altitude, (**b**) Graph showing the occurrence of the 19 amphibian and 20 reptile species according to habitat type.

Herpetofauna species were most commonly distributed at an altitude of 168.00 m (first–third quartiles: 65.00–319.00 m); specifically, amphibians were most commonly distributed at an altitude of 168.00 m (first–third quartiles: 61.00–279.00 m) and reptiles at an altitude of 153.00 m (first–third quartiles: 61.00–279.00 m). Generally, compared to reptiles, amphibians were distributed at lower altitudes (Fig. 1a). The habitat type where amphibians were observed the most commonly was farmland (50.94%), followed by forests (47.92%), grasslands (1.11%), and urban areas (0.03%), whereas reptiles were observed the most commonly in forests (47.92%), followed by farmland (44.58%), grassland (2.18%), and urban areas (0.04%) (Fig. 1b).

### Species distribution model

The average AUC of the 19 amphibian and 20 reptile species was 0.798 ± 0.139 and 0.764 ± 0.103, respectively. The 10% training error value of the test sample was 0.120 ± 0.020 for amphibians and 0.180 ± 0.074 for reptiles, indicating that the overall error was low (Table 2).

**Table 2:** Summary of species distribution models for the 19 amphibian and 20 reptilian species using Maxent modeling.

Order	Species	AUC		Logistic threshold	Omission		Contributing variable		
		Training value	Test value		Training value	Test value	1st	2nd	3rd
**Amphibia**									
Caudata									
	*Hynobius leechii*	0.647	0.635	0.462	0.100	0.103	Altitude	Bio2	Bio14
	*Hynobius yangi*	0.995	0.995	0.319	0.067	0.093	Bio2	Bio13	Bio12
	*Hynobius quelpaertensis*	0.969	0.961	0.094	0.100	0.138	Bio2	Bio14	Bio1
	*Hynobius unisacculus*	0.983	0.978	0.209	0.095	0.152	Bio12	Bio1	Bio2
	*Onychodactylus koreanus*	0.877	0.855	0.289	0.097	0.143	Land	Bio1	Altitude
	*Karsenia koreana*	0.967	0.957	0.164	0.100	0.165	Bio14	Bio2	Bio13
**Anura**									
	*Bombina orientalis*	0.687	0.678	0.458	0.100	0.111	Altitude	Bio1	Bio3
	*Bufo gargarizans*	0.669	0.646	0.471	0.099	0.116	Altitude	Bio1	Bio2
	*Bufo stejnegeri*	0.910	0.889	0.346	0.095	0.114	Bio1	Altitude	Land
	*Dryophytes japonica*	0.620	0.609	0.514	0.100	0.107	Altitude	Bio1	Bio14
	*Dryophytes suweonensis*	0.973	0.967	0.300	0.085	0.138	Altitude	Bio13	Bio1
	*Kaloula borealis*	0.879	0.836	0.263	0.092	0.149	Altitude	Bio2	Bio1
	*Pelophylax nigromaculatus*	0.620	0.615	0.480	0.100	0.100	Altitude	Bio1	Bio13
	*Pelophylax chosenicus*	0.928	0.908	0.225	0.087	0.116	Altitude	Bio1	Bio13
	*Rana coreana*	0.726	0.715	0.353	0.100	0.102	Altitude	Bio1	Bio12
	*Rana uenoi*	0.639	0.632	0.459	0.100	0.108	Altitude	Bio14	Bio2
	*Rana huanrenensis*	0.825	0.817	0.333	0.100	0.113	Altitude	Bio1	Land
	*Glandirana rugosa*	0.673	0.659	0.422	0.100	0.111	Altitude	Bio2	Bio1
	*Lithobates catesbeianus*	0.814	0.813	0.341	0.100	0.099	Land	Bio1	Altitude
	Average ± S.D	0.810 ± 0.138	0.798 ± 0.139	0.342 ± 0.115	0.096 ± 0.008	0.120 ± 0.020			
**Reptilia**									
**Testudinata**									
	*Pelodiscus maackii*	0.840	0.731	0.288	0.091	0.248	Bio3	Bio14	Altitude
	*Mauremys reevesii*	0.869	0.746	0.357	0.095	0.362	Altitude	Bio14	Bio12
	*Trachemys scripta elegans*	0.847	0.811	0.238	0.090	0.172	Altitude	Bio14	Bio1
**Squamata (Lacertilia)**									
	*Gekko japonicus*	0.987	0.973	0.202	0.095	0.267	Bio1	Bio2	Bio12
	*Scincella vandenburghi*	0.819	0.798	0.276	0.098	0.115	Bio2	Bio13	Bio1
	*Scincella huanrenensis*	0.964	0.932	0.320	0.091	0.200	Land	Bio13	Bio1
	*Takydromus amurensis*	0.749	0.729	0.306	0.099	0.122	Land	Altitude	Bio2
	*Takydromus wolteri*	0.768	0.756	0.294	0.100	0.106	Bio2	Bio1	Bio14
	*Eremias argus*	0.904	0.799	0.117	0.067	0.240	Altitude	Bio14	Bio1
**Squamata (Serpentes)**									
	*Oocatochus rufodorsatus*	0.744	0.707	0.315	0.099	0.131	Altitude	Bio14	Bio1
	*Elaphe dione*	0.665	0.636	0.375	0.099	0.127	Altitude	Bio2	Bio13
	*Elaphe schrenckii*	0.789	0.720	0.269	0.091	0.146	Bio14	Bio3	Altitude
	*Rhabdophis tigrinus*	0.622	0.609	0.408	0.100	0.104	Altitude	Bio12	Bio2
	*Hebius vibakari*	0.996	0.994	0.349	0.056	0.333	Bio2	Bio12	Altitude
	*Sibynophis chinensis*	0.862	0.805	0.116	0.100	0.158	Bio14	Bio2	Bio12
	*Lycodon rufozonatus*	0.706	0.663	0.365	0.100	0.122	Altitude	Bio2	Bio13
	*Orientocoluber spinalis*	0.878	0.777	0.091	0.083	0.217	Bio2	Altitude	Bio13
	*Gloydius ussuriensis*	0.671	0.655	0.373	0.100	0.114	Altitude	Bio2	Bio13
	*Gloydius brevicaudus*	0.691	0.660	0.381	0.100	0.135	Altitude	Bio1	Bio2
	*Gloydius intermedius*	0.812	0.771	0.302	0.093	0.175	Altitude	Bio1	Land
	Average ± S.D	0.809 ± 0.106	0.764 ± 0.103	0.287 ± 0.090	0.092 ± 0.011	0.180 ± 0.074			

**Figure 2:**
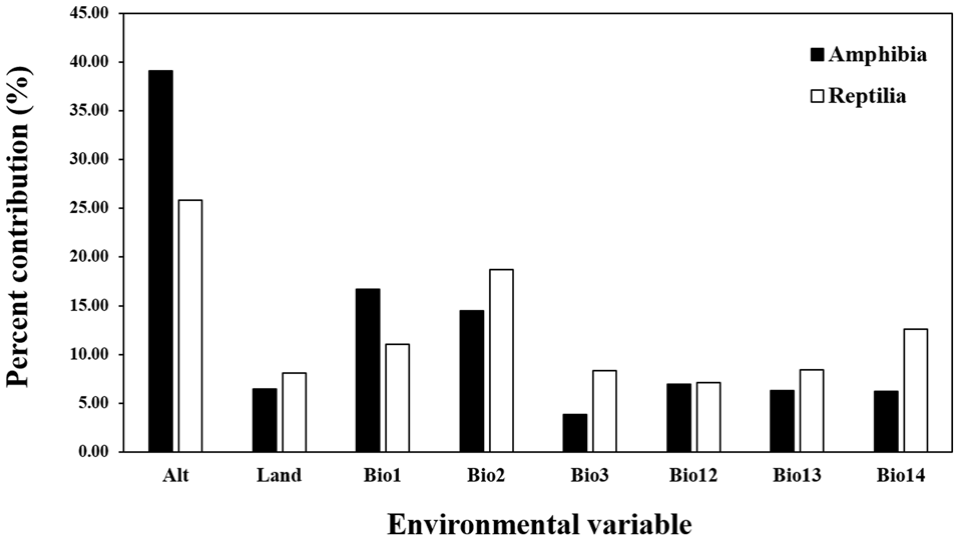
Percent contribution (%) of environmental variables to the species distribution model for the 19 amphibian and 20 reptile species. The percentage contribution shows the importance of variables determined by the jackknife test. *Alt* altitude (m), *Land* land cover, *Bio1* annual temperature (°C), *Bio2* mean diurnal range (°C), *Bio3* isothermality (standard deviation × 100; °C), *Bio12* annual precipitation (mm), *Bio13* precipitation in the wettest period (mm), *Bio14* precipitation in the driest period (mm).

The variable with the highest contribution in the distribution model of amphibians was altitude (39.10%), followed by the variables Bio1 (16.71%) and Bio4 (14.46%) (Fig. 2). Altitude showed the highest contribution in 12 out of 19 models (Table 2). The variable with the highest contribution in the distribution model of reptiles was also altitude (25.79%), followed by the variables Bio2 (18.67%) and Bio1 (11.03%) (Fig. 2). Altitude showed the highest contribution in 10 out of 20 models (Table 2).

### Main distribution areas

**Figure 3:**
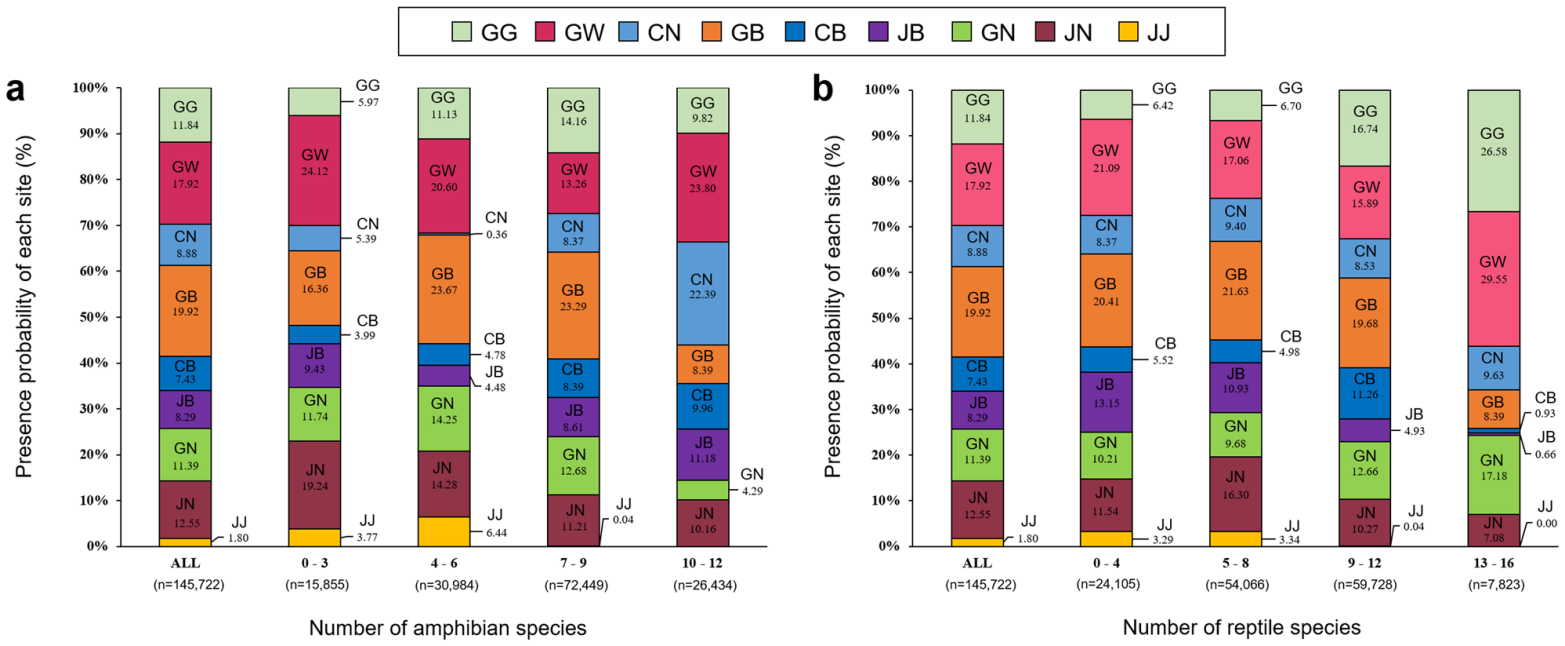
Percentage distribution of each province according to the number of cells in which the presence of (**a**) the 19 amphibian species and (**b**) the 20 reptile species were predicted. Province abbreviations: *GG* Gyeonggi, *GW* Gangwon, *CN* Chungnam, *CB* Chungbuk, *GB* Gyeongbuk, *JB* Jeonbuk, *GN* Gyeongnam, *JN* Jeonnam, *JJ* Jeju.

**Figure 4:**
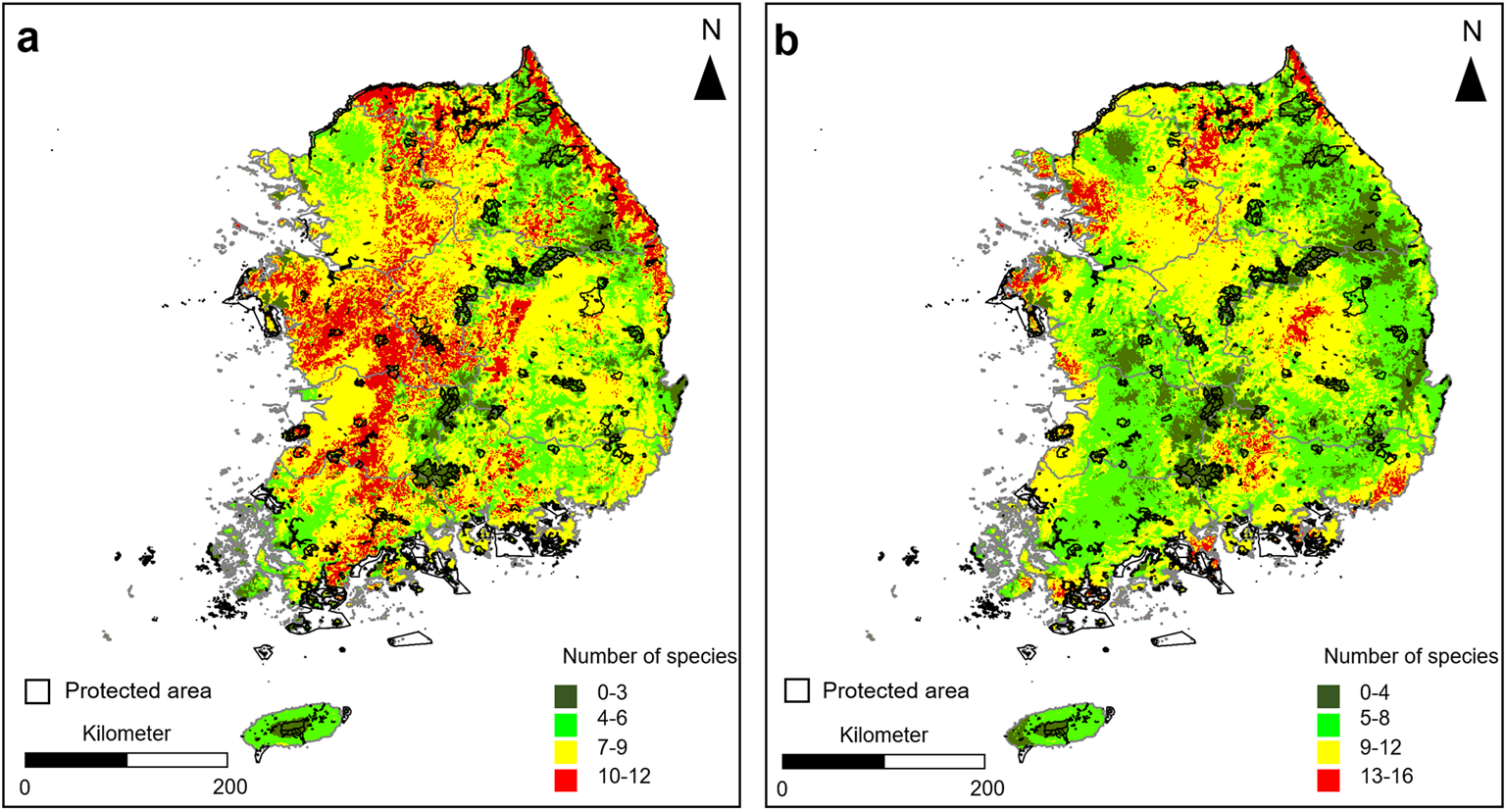
The overlap of predicted presence/absence maps of (**a**) the 19 amphibian species and (**b**) the reptile 20 species in South Korea. This map was generated using the tool of ArcGIS 10.3 (ESRI, Redlands, CA, USA, http://www.esri.com).

Herpetofauna species were predicted to be distributed in most areas of South Korea. For amphibians, the areas where 7–9 species coexisted were predicted to be the most with 72,449 cells, and for reptiles, the areas where 9–12 species coexisted were predicted to be the most with 59,728 cells. The hotspot areas for amphibians, where the most diverse species coexisted, accounted for 26,434 cells with 10–12 species, and the hotspot areas for reptiles accounted for 7,823 cells with 13–16 species (Figs. 3a,b, 4a,b). The hotspot areas of amphibians included Gangwon-do (23.80%), Chungcheongnam-do (22.39%), and Jeollabuk-do (11.18%), and the core distribution areas of reptiles included Gangwon-do (29.55%), Gyeonggi-do (26.58%), and Gyeongsangnam-do (17.18%) (Figs. 3a,b, 4a,b).

**Figure 5:**
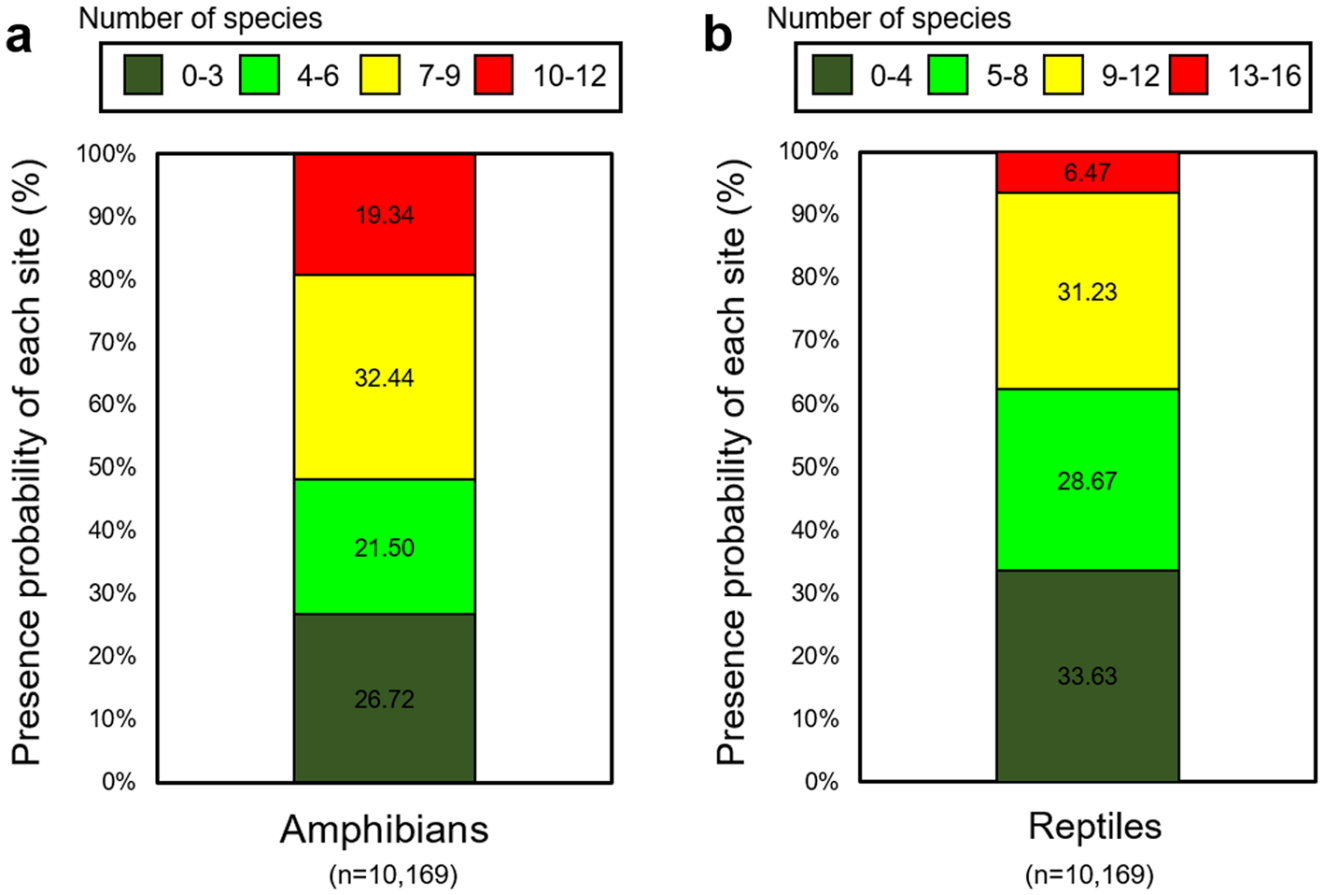
The ecological conservation area ranges and overlap of predicted presence/absence distributions of (**a**) amphibian and (**b**) reptile species in South Korea. This figure was generated using the program of Microsoft Excel and PowerPoint (MS, Microsoft Office 2016, WA, USA, https://www.microsoft.com).

In the 10,169 cells designated as national conservation areas, the areas where 7–9 amphibian species coexisted were predicted to account for the most, at 32.44%, while the areas where 9–12 reptile species coexisted were predicted to account for the most, at 31.23%. Hotspot areas in the national conservation areas accounted for 19.34% for amphibians and 6.47% for reptiles (Fig. 5).

## Discussion

In the present study, major habitats of herpetofauna species were predicted using distribution models of 19 amphibian and 20 reptile species inhabiting South Korea. A high correlation was identified between important climatic variables in the areas where herpetofauna species were distributed and geographical variables, and altitude was found to be an environmental variable with the most influence on their distribution. Hotspot area predictions showed that the province with the most diverse species was Gangwon-do, around the Taebaek Mountains.

Altitude was an important environmental factor affecting the distribution of herpetofauna species, showing a high contribution in most models. In general, different herpetofauna species are distributed at different altitudes because of their limited home ranges and habitation environments, and altitude, among various environmental variables, is known to have a major influence on their distribution^7,27,29,30^. The preferred altitude range is known to vary among the herpetofauna species distributed in South Korea (Supplementary Table S2)^7,25^. For example, while most of the 19 amphibian species are mainly distributed at altitudes between 0 and 500 m, three species, namely *Kaloula borealis*, *Rana plancyi*, and *Hyla suwonesis*, are known to prefer low altitudes between 0 and 100 m, whereas three species, namely *Bufo stejnegeri*, *Onychodactylus fischeri*, and *Rana huanrenensis*, are known to prefer high altitudes between 400 and 700 m^25,30,35^. Most of the 20 reptile species are mainly distributed at altitudes between 0 and 500 m, except for the following three species: *Eremias argus*, which is known to prefer low altitudes between 0 and 100 m, and *Gloydius intermedius* and *Scincella huanrenensis*, which are known to prefer altitudes higher than 400 m^7,29,34^. The results of the present study were consistent with the elevation distributions for herpetofauna species reported in previous studies.

We found that herpetofauna species distributed in South Korea preferred forests and farmland, with amphibians more commonly inhabiting farmland and reptiles more commonly inhabiting forests (Fig. 1b). Paddy wetlands provide an essential aquatic environment for skin-breathing amphibians, and 16 of the 19 amphibian species inhabiting South Korea, except for *Kaloula borealis*, *Rana plancyi*, and *Karsenia koreana*, use paddy wetlands as breeding grounds^28,30,36–38^. Furthermore, among these 16 amphibian species, all but two species, *Rana plancyi* and *Hyla suwonesis*, are known to prefer paddy wetlands located near mountainous areas rather than plains, and their population size is also known to be larger than that of the other two species^27,30,38,39^. Among the 20 reptile species, all but two species, *Eremias argus* and *Gekko japonicus*, prefer forests, using rivers, valleys, ridges, grasslands, wetlands, and other habitats in the surrounding areas for breeding and hibernation^29,34,40–44^. Furthermore, 18 out of 20 reptile species, excluding *Gloydius intermedius* and *Scincella huanrenensis*, appear in paddy wetlands, which they use for foraging^29,34,40^. Therefore, forests and agricultural land, which were the main habitats of herpetofauna species identified from a macroscopic point of view, adequately reflected the main habitats of herpetofauna species reported in previous studies. The most important habitats were paddy wetlands for amphibians and forests for reptiles^27,31^.

The geographic distributions of herpetofauna species predicted by species distribution modeling were consistent with the geographic ranges reported by previous surveys (Supplementary Figs. S1, S2). According to previous studies, nine amphibian species are widely observed inland, while the others have a limited distribution range^24,30,33^. *Onychodactylus fischeri, Bufo stejnegeri*, and *Rana huanrenensis* are densely distributed in the northeastern regions, including Gangwon-do, Gyeonggi-do, and Gyeongsangbuk-do. *Kaloula borealis*, *Rana plancyi*, and *Hyla suwonesis* are found in central and western regions, including Chungcheongnam-do and Gyeonggi-do, while *Karsenia koreana* is found in the central regions of Chungcheongnam-do and Chungcheongbuk-do. *Rana catesbeiana* is found in southern regions, including Gyeongsangnam-do, Jeollanam-do, and Jeju-do, and *Hynobius yangi* is concentrated in Gyeongsangnam-do^26,39,40,45^. Regarding reptiles, 13 species are widely observed inland, while *Scincella huanrenensis* and *Gloydius intermedius* are mainly distributed in northeastern regions such as Gangwon-do, Gyeonggi-do, and Gyeongsangbuk-do; *Eremias argus* in central and western regions such as Chungcheongnam-do and Gyeonggi-do; *and Gekko japonicus*, *Sibynophis chinensis*, *Coluber spinalis*, and *Amphiesma vibakari ruthveni* in southern regions such as Gyeongsangnam-do, Jeollanam-do, and Jeju-do^40,46,47^.

Recent studies have indicated the need for using habitat prediction models to establish protected areas for wild animals and plants in South Korea^5,33,48,49^. Habitat prediction model studies can be used to provide objective and scientific methods and procedures for the establishment of protected areas^4,6,20^. For example, hotspot areas were identified, and protected areas were established or proposed for 16 *Hylidae* species that inhabited South America, 7 *Viperidae* species in Africa, and all herpetofauna species in Madagascar and Morocco^4,6,20,50^. The main distribution areas of the seven endangered herpetofauna species in South Korea were found to be Chungcheong-do and western Gyeonggi-do, and the main distribution areas of three amphibian species of the genus *Rana* and three reptile species of the genus *Gloydius* are located around the Taebaek Mountains in Gangwon-do^7,29,30^. These areas were similar in location and extent to the hotspot areas identified in the present study and included national conservation areas such as national parks. Nevertheless, some modifications should be made, and additional conservation areas should be established considering the hotspot areas where many herpetofauna species were observed.

In the present study, additional hotspot areas for herpetofauna species were predicted to be located in Gangwon-do than in the other provinces (Fig. 3). In Gangwon-do, forests account for 81% of the total area, mostly because of the presence of the Taebaek Mountains. As Gangwon-do is the administrative district with the lowest population density, various ecological conservation areas designated by the government are located here^51,52^. The national conservation areas in Gangwon-do, where many of the hotspot areas for herpetofauna species are located, include the Demilitarized Zone (DMZ) located in the northern region; the water resources conservation areas of Chuncheonho Lake, Paroho Lake, and Soyangho Lake located in the western region; and Seoraksan and Odaesan National Parks located in the eastern region (Fig. 4). In these areas, the ecosystem is not damaged, and high biodiversity is maintained as access by civilians has been restricted since the end of the Korean War in 1953^53,54^. According to previous surveys conducted in the DMZ from 1989 to 2016, 16 amphibian and 18 reptile species were known to inhabit the area^55^. The habitat status data collected from 1997 to 2019 showed 8–12 amphibian species and 10–14 reptile species in the national parks located in Gangwon-do^56^. Additionally, we identified hotspot areas not only in the conservation areas but also in other areas within Gangwon-do due to its low population density and well-conserved ecological environments.

In the present study, the hotspot areas for herpetofauna species were identified in various regions other than Gangwon-do. In particular, hotspot areas for amphibians were mostly concentrated in Chungcheongnam-do and its surrounding areas, and hotspot areas for reptiles were concentrated in the areas around the southwest coast and the areas of the four major rivers (Fig. 4). Chungcheongnam-do and its surrounding areas consist of plains with low altitudes. In this province, well-developed paddy wetlands are used by amphibians as their main feeding and breeding grounds. Unlike other regions, this province has high biodiversity, with dense populations of endangered amphibians such as *Kaloula borealis*, *Rana plancyi*, and *Hyla suwonesis*^7,27^. In the areas around the southwest coast and the areas of the four major rivers (Hangang River, Geumgang River, Nakdonggang River, and Yeongsangang River), high biodiversity may have been identified because of the habitats of *Eremias argus*, *Pelodiscus sinensis, Chinemys reevesii*, and *Trachemys scripta elegans*, which mainly inhabit coastal sand dunes, rivers, and streams, which are not present in other regions^7,40,57^. However, many of the hotspot areas for amphibians were not included in the national conservation areas.

The national conservation areas of South Korea included more than 30% of areas inhabited by 7–9 amphibian species and nine to 13 reptile species together, as well as some hotspot areas for herpetofauna species, playing an important role in habitat conservation. However, more hotspot areas were identified outside the national conservation areas. Therefore, we showed that habitat protection is not carried out in these hotspot areas not included in the national conservation areas, with a high risk of habitat destruction because of development activities such as road and apartment construction. Therefore, to protect herpetofauna species, it is necessary to establish new conservation areas focusing on herpetofauna species after confirming the actual inhabitation of species through precise monitoring in the predicted hotspot areas. Furthermore, the hotspot areas where the actual habitats were identified need to be designated as protected areas with priority over other areas by restricting development, tree harvesting, and the inflow of farms. These results can serve as important basic data for establishing protection measures and designating protected areas for herpetofauna species.

A comprehensive analysis of the hotspot areas of 39 herpetofauna species revealed that Gangwon-do was the province with the highest number of hotspot areas, predicted to be inhabited by 10–12 amphibian species and 13–16 reptile species. In addition, amphibians were predicted to be concentrated in paddy wetlands around Chungcheongnam-do, and reptiles in the areas around the southwestern coast and areas of the four major rivers. Some hotspot areas were included within the national conservation areas, but many hotspot areas were located in areas not designated as conservation areas. It is necessary to protect the habitats of herpetofauna species by expanding the conservation areas after verification through detailed surveys in these areas. In the future, if the exact distribution range of *Dryophytes flaviventris*, a recently discovered species not included in this study, is revealed, more hotspot areas in addition to those revealed in this study may be discovered.

## Methods

### Study area

**Figure 6:**
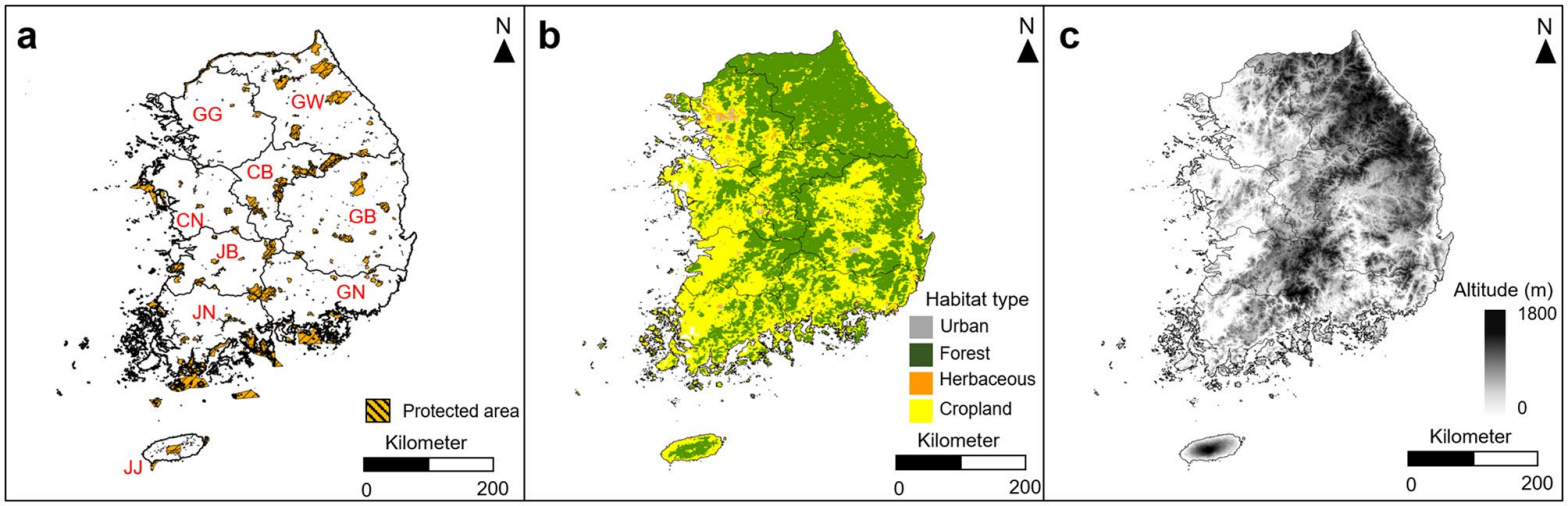
Topographic maps of (**a**) protected areas, (**b**) habitat types, (**c**) altitudes in South Korea. Province abbreviations: *GG* Gyeonggi, *GW* Gangwon, *CN* Chungnam, *CB* Chungbuk, *GB* Gyeongbuk, *JB* Jeonbuk, *GN* Gyeongnam, *JN* Jeonnam, *JJ* Jeju. This map was generated using the tool of ArcGIS 10.3 (ESRI, Redlands, CA, USA, http://www.esri.com).

The study was conducted in South Korea, covering several regions of the Korean Peninsula and several islands, including Jeju Island. About 70% of the investigated area was covered with forests, and about 30% was agricultural land. The eastern region has a high altitude because of the Taebaek Mountains, whereas the western region is characterized by low-altitude terrain with plains and arable land (Fig. 6b,c). Korea has a continental climate with four distinct seasons, with cold and dry winters and hot and humid summers. It is divided into nine provinces: Gyeonggi-do (GG), Gangwon-do (GW), Chungcheongnam-do (CN), Chungcheongbuk-do (CB), Gyeongsangnam-do (GN), Gyeongsangbuk-do (GB), Jeollanam-do (JN), Jeollabuk-do (JB), and Jeju Island (JJ) (Fig. 6a).

### Species distribution data and environment analysis

The observation data of herpetofauna species inhabiting South Korea was obtained from three sources: data from the National Natural Environment Survey conducted by the National Institute of Ecology and the National Academy of Environmental Sciences (Ref.^58^; survey period: 2005–2017), natural resource survey data provided by the Korea National Park Research Institute (Ref.^59^; survey period: 2004–2011), and data provided by the Global Biodiversity Information Facility (Ref.^60^; observation period: 2004–2019). For *Gekko japonicus* and *Sibynophis chinensis*, which lacked observation points, the observation points used in previous studies were used in the present study as well^32,61^ (Table 1). The location of species observed in most of South Korea (98.8% of the total land area, or 99,000 km^2^) could be confirmed based on the results of the surveys described above. All applied data were collected through field surveys by herpetofauna experts with over ten years of experience. The survey period was from early spring (February) to early winter (November), when reptiles and amphibians are active in South Korea. Its geographic scope covered the entire country, including the land and many islands^40^. All experts visually identified the species of individuals detected while walking or traveling in a car and collected geographical information. A total of 19 amphibian species and 20 reptile species were used for the analysis, excluding *Dryophytes flaviventris*, which was recently identified to inhabit South Korea^62^.

The environmental variables used to identify the main distribution areas of amphibians and reptiles included altitude and climate data (six out of 19 variables) obtained from a 1:25,000 scale level 2 land cover map^63^ and Worldclim v. 1.4^64^ (Table 3). All grids were of a uniform size of 30′′ (about 1 km^2^). In order to identify the types of habitats preferred by the studied species, the land cover map was divided into four habitat types by determining similar or overlapping variables (Supplementary Table S1). Since climate variables are highly correlated with each other, the variables with high correlation (Pearson's correlation coefficients (r) > 0.8) were excluded from the analysis to minimize the effect of multicollinearity^35,65,66^. Accordingly, the following six climate variables were used in the present study: annual average temperature (Bio1), average diurnal temperature range (Bio2), isotherm (Bio3), annual average precipitation (Bio12), summer precipitation (Bio13), and winter precipitation (Bio14). The distribution points of the herpetofauna species were projected onto all environmental variables, and the habitat environment was checked using the extracted values, and a kernel probability density plot was generated for the altitude values. According to data normality, all data were expressed as means with standard deviations, medians, or first–third quartiles. Statistical analysis was performed using R version 3.0.2^67^.

**Table 3:** Environmental variables used for Maxent modeling the distribution of the 19 amphibian and 20 reptile species in South Korea.

Code	Variable	Type	Range in South Korea	Amphibia range	Reptilia range
Alt	Altitude (m)	Continuous	0.00–1817.00	0.00–1232.00	0.00–1232.00
Land	Land cover	Categorical	15 categories	Four categories	Four categories
Bio1	Annual temperature (°C)	Continuous	2.30–16.00	4.50–16.00	5.00–16.00
Bio2	Mean diurnal range (°C)	Continuous	6.30–12.90	6.30–12.50	6.30–12.50
Bio3	Isothermality (standard deviation × 100; °C)	Continuous	2.10–3.30	2.10–3.30	2.10–3.30
Bio12	Annual precipitation (mm)	Continuous	948.00–2137.00	997.00–1851.00	978.00–1806.00
Bio13	Precipitation in the wettest period (mm)	Continuous	143.00–462.00	174.00–460.00	156.00–458.00
Bio14	Precipitation in the driest period (mm)	Continuous	15.00–57.00	15.00–51.00	15.00–51.00

Data^39^ (http://www.worldclim.com).

### Species distribution modeling

The maximum entropy approach model (Maxent version 3.3.3 k)^68^, one of the species distribution model (SDM) algorithms, is the most widely used for wild organisms and provides the highest prediction result based on regression analysis^33,69,70^ Unlike other algorithms (e.g., GLM, GAM, RF, etc.), this model integrates pseudo-absence points without any assumption of certainty and maintains the most possible uniform distribution under the limitations imposed by predictor variables, leading to the least bias for the presence of predicted results and its most conservative estimates^71–74^. Since Maxent can predict even with a small number of samples due to the use of appearance data alone, it is actively used in studies on reptiles and amphibians that are difficult for field observation^29,75–78^. An SDM was constructed using the appearance data of reptiles and amphibians as dependent variables and environmental variables (a total of eight environmental variables; Table 3) as independent variables. The models were repeatedly run 15 times using default parameters, including logistic output, 1 for regularization multiplier, and 10,000 for background points^29,79^. This study generated a potential dichotomous (presence/absence) distribution raster based on 10% training presence (including 90% of occurrences) as the threshold^7–10^. The 10th percentile threshold has the advantages of being less sensitive to extreme environmental values and reducing commission errors^80,81^. Thus, it is used for wild animals, including amphibians and reptiles that are mobile^77,78,82,83^. To evaluate the model, the dataset was divided into a training set for 75% and a testing set for 25% through the random test percentage, and subjected to 5000 iterations^29,79^. The explanatory power of the model was verified by calculating the area under the curve (AUC), which is the value of the lower area of the curve, by receiver operating characteristics (ROC) verification. AUC values range from 0.0 to 1.0, with a value closer to 1.0 indicating a higher prediction accuracy of the model^84^. Most studies using habitat prediction programs used AUC values to evaluate model performance, which can be sensitively affected by model conditions such as the number of samples and resolution^85–89^. In order to supplement this, some studies related to SDM suggest an omission rate in addition to the AUC value^10,30,79^. The omission rate is calculated as the ratio of points that were not predicted based on a threshold and were thus missing. The values range from 0.0 to 1.0, with a lower value indicating fewer omissions in the analysis process. Therefore, in the present study, besides the AUC value, the omission rate shown in the 10% training presence was also considered^86^. The contribution of each environmental variable to the areas where the herpetofauna species were distributed was calculated using the average percent contribution determined by the jackknife test.

The derived SDMs were overlapped based on species through Arc GIS (Ver. 10.3; ESRI, Redlands, CA, USA), and the number of cells present in each of the nine administrative areas was identified by a percentage based on interspecies overlapping areas. Furthermore, by superimposing the generated species distribution map, the geographic range of the hotspot areas where herpetofauna species were densely distributed was identified. The areas predicted to be inhabited by 10–12 amphibian species and those predicted to be inhabited by 13–16 reptile species were determined as hotspot areas^90,91^. To identify the existing national conservation areas designated in South Korea, the geographic scope of the environmental conservation areas and national parks obtained from the National Geographic Information Institute was used (Fig. 6a)^52,92–94^.

## Supplementary Information

Supplementary Information.

## Data Availability

The datasets generated and/or analyzed during the current study are available from the corresponding author on reasonable request.
