# Supplementary material for: The establishment of ecological conservation for herpetofauna species in hotspot areas of South Korea
Source: Sci Rep. 2022 Sep 1;12:14839. doi: 10.1038/s41598-022-19129-0 (PMC9436999; doi:10.1038/s41598-022-19129-0)

**Supplementary Information**

**How to Conserve the Hotspot Areas for Herpetofauna Species in South Korea?**

Min Seock Do^1^, Seok-Jun Son^2^, Green Choi^3^, Nakyung Yoo^4^, Dae-in Kim^5^, Kyo-Soung Koo^6^, Hyung-Kyu Nam^1*^

^1^ National Institute of Biological Resources, Seo-gu Incheon 22689, South Korea

^2^ Korea Institute of Wildlife Ecology, Daejeon 34388, Republic of Korea

^3^ MEET GREEN, Seocheon 33646, Republic of Korea

^4^ National Institute of Ecology, Yeongyang 36541, Republic of Korea

^5^ HERPING, Seoul 02505, Republic of Korea

^6^ Interdisciplinary Program of EcoCreative, Ewha Woman's University, Seoul 07804, Republic of Korea

*Corresponding author: Hyung-Kyu Nam

Phone: +82-32-590-7239; Fax: +82-32-590-3867;

E-mail: [namhk2703@korea.kr](mailto:namhk2703@korea.kr)

ORCID No. 0000-0002-9619-2478

**Supplementary Table S1**

The land cover types according to the National Geographic Information Institute (NGII) and those used in the present study. The distribution of Korean species of reptiles and amphibians, which is classified into fourteen habitat types according to NGII, was classified into four habitat types in the present study.

| Land cover type | Type used in the present study |
| --- | --- |
| Broadleaf Deciduous Forest | Forest |
| Needleleaf Evergreen Forest |  |
| Needleleaf Deciduous Forest |  |
|  |  |
| Herbaceous | Herbaceous |
| Herbaceous with Sparse Trees |  |
| Sparse Herbaceous |  |
| Bare and Grass Land |  |
|  |  |
| Cropland | Cropland |
| Farm Area |  |
| Rice Paddy |  |
|  |  |
| Residential Area | Urban |
| Commercial Area |  |
| Manufacturing Area |  |
| Urban |  |

**Supplementary Table S2**

Median (1^st^–3^rd^ quartiles) values of environmental variable ranges (climate and altitude) in which the 19 amphibian and 20 reptile species were identified

|  | Altitude | Bio1 | Bio2 | Bio3 | Bio12 | Bio13 | Bio14 |
| --- | --- | --- | --- | --- | --- | --- | --- |
| Amphibia |  |  |  |  |  |  |  |
| *Hynobius leechii* | 183.50 (94.25-306.00) | 11.60 (10.60-12.60) | 10.80 (9.70-11.30) | 2.90 (2.70-3.00) | 1303.00 (1223.00-1395.75) | 285.00 (253.00-312.00) | 27.00 (23.00-32.00) |
| *Hynobius yangi* | 15.00 (5.00-73.00) | 14.20 (13.80-14.20) | 8.60 (8.40-8.60) | 2.70 (2.60-2.70) | 1316.00 (1301.00-1333.00) | 216.00 (210.00-220.00) | 30.00 (29.00-31.00) |
| *Hynobius quelpaertensis* | 157.00 (98.25-310.75) | 13.65 (12.83-14.70) | 6.80 (6.60-8.88) | 2.50 (2.40-2.60) | 1522.50 (1352.00-1670.25) | 251.50 (235.25-270.00) | 44.00 (36.00-47.00) |
| *Hynobius unisacculus* | 158.00 (59.00-171.00) | 13.40 (13.30-13.98) | 8.90 (8.50-9.40) | 2.70 (2.70-2.80) | 1576.00 (1576.00-1633.50) | 279.50 (272.00-297.75) | 28.00 (27.00-30.00) |
| *Onychodactylus koreanus* | 474.00 (309.75-634.75) | 9.10 (8.00-10.10) | 11.10 (10.40-11.40) | 2.80 (2.80-2.90) | 1402.00 (1350.25-1459.00) | 327.00 (304.00-353.00) | 30.00 (23.00-36.00) |
| *Karsenia koreana* | 320.00 (233.00-410.00) | 10.90 (10.30-11.40) | 11.20 (11.10-11.20) | 2.90 (2.90-3.00) | 1334.00 (1309.00-1353.00) | 311.00 (302.00-318.00) | 31.00 (30.00-32.00) |
| *Bombina orientalis* | 217.00 (129.00-348.50) | 11.70 (10.40-12.90) | 10.70 (9.80-11.20) | 2.90 (2.80-3.00) | 1339.00 (1222.00-1460.50) | 285.00 (249.00-312.00) | 26.00 (22.00-31.00) |
| *Bufo gargarizans* | 140.00 (57.00-244.50) | 11.90 (11.20-13.00) | 10.70 (9.60-11.30) | 2.90 (2.70-3.00) | 1286.00 (1203.75-1397.00) | 282.00 (255.00-306.00) | 26.00 (22.00-31.00) |
| *Bufo stejnegeri* | 402.00 (270.50-578.00) | 9.30 (8.20-10.20) | 10.80 (10.15-11.30) | 2.80 (2.70-2.85) | 1387.00 (1323.50-1460.00) | 331.00 (290.50-370.00) | 29.00 (22.00-37.00) |
| *Dryophytes japonica* | 129.00 (47.00-240.00) | 11.80 (11.00-12.90) | 10.60 (9.50-11.20) | 2.80 (2.70-3.00) | 1286.00 (1217.00-1411.00) | 286.00 (259.00-316.00) | 26.00 (22.00-31.00) |
| *Dryophytes suweonensis* | 8.50 (6.00-28.25) | 11.80 (11.70-12.08) | 10.50 (9.80-10.90) | 2.70 (2.70-2.80) | 1237.50 (1212.00-1248.75) | 303.00 (275.25-324.75) | 24.00 (23.00-28.00) |
| *Kaloula borealis* | 27.50 (13.00-90.50) | 12.00 (11.70-12.70) | 9.70 (8.90-10.40) | 2.70 (2.50-2.80) | 1245.00 (1187.00-1308.25) | 269.50 (257.00-300.00) | 28.00 (24.75-32.00) |
| *Pelophylax nigromaculatus* | 110.00 (38.00-221.00) | 12.00 (11.20-13.00) | 10.40 (9.40-11.20) | 2.80 (2.70-3.00) | 1286.00 (1216.00-1411.00) | 284.00 (259.00-310.00) | 26.00 (23.00-31.00) |
| *Pelophylax chosenicus* | 17.00 (7.00-44.50) | 11.90 (11.70-12.30) | 9.30 (8.90-10.65) | 2.60 (2.50-2.80) | 1215.00 (1183.00-1271.00) | 283.00 (261.00-299.50) | 27.00 (22.00-29.00) |
| *Rana coreana* | 101.00 (45.00-190.25) | 11.70 (11.20-12.30) | 10.60 (9.70-11.20) | 2.80 (2.70-2.90) | 1262.00 (1213.00-1320.25) | 287.00 (261.00-308.25) | 27.00 (23.00-31.00) |
| *Rana uenoi* | 212.00 (121.00-342.50) | 11.40 (10.30-12.50) | 10.70 (9.80-11.20) | 2.90 (2.70-2.90) | 1313.00 (1230.00-1420.00) | 287.00 (258.00-321.00) | 27.00 (23.00-33.00) |
| *Rana huanrenensis* | 414.00 (270.00-571.00) | 9.70 (8.50-11.00) | 10.80 (10.00-11.20) | 2.80 (2.80-2.90) | 1369.00 (1297.00-1448.00) | 309.00 (276.00-334.00) | 31.00 (25.00-37.00) |
| *Glandirana rugosa* | 194.00 (111.00-305.00) | 11.40 (10.60-12.20) | 10.90 (10.10-11.30) | 2.90 (2.70-3.00) | 1306.00 (1223.00-1414.00) | 296.00 (263.00-324.00) | 26.00 (22.00-30.00) |
| *Lithobates catesbeianus* | 51.00 (13.00-120.75) | 13.00 (12.40-13.60) | 10.00 (9.10-11.00) | 2.80 (2.60-3.00) | 1264.00 (1197.25-1382.00) | 268.00 (247.00-287.00) | 28.00 (23.00-34.00) |
| Reptilia |  |  |  |  |  |  |  |
| *Pelodiscus maackii* | 117.50 (53.75-223.25) | 11.95 (11.10-12.40) | 11.25 (10.00-11.50) | 2.80 (2.78-3.00) | 1303.50 (1239.50-1353.00) | 297.00 (271.00-319.25) | 24.00 (21.00-27.25) |
| *Mauremys reevesii* | 111.50 (67.75-192.00) | 12.05 (11.08-13.20) | 11.10 (10.35-11.60) | 2.90 (2.80-3.03) | 1316.00 (1258.00-1379.00) | 293.50 (268.50-316.75) | 23.50 (20.75-28) |
| *Trachemys scripta elegans* | 61.00 (23.50-156.00) | 12.70 (11.80-13.60) | 10.80 (9.10-11.20) | 2.80 (2.60-3.00) | 1308.00 (1249.00-1375.50) | 280.00 (249.50-301.50) | 23.00 (21.00-28.50) |
| *Gekko japonicus* | 6.00 (4.00-22.00) | 14.30 (14.20-14.60) | 8.00 (8.00-8.90) | 2.60 (2.50-2.70) | 1412.00 (1145.00-1416.00) | 242.00 (204.00-243.00) | 29.00 (29.00-31.00) |
| *Scincella vandenburghi* | 124.00 (44.00-238.00) | 12.80 (11.30-13.90) | 9.50 (8.40-11.20) | 2.70 (2.50-2.90) | 1344.00 (1240.00-1531.00) | 278.00 (265.00-300.00) | 28.00 (25.00-37.00) |
| *Scincella huanrenensis* | 543.50 (248.50-753.25) | 8.20 (7.10-9.88) | 10.75 (10.23-11.00) | 2.70 (2.70-2.80) | 1454.50 (1418.25-1506.50) | 413.00 (344.75-425.75) | 23.50 (19.25-36.00) |
| *Takydromus amurensis* | 269.00 (132.00-473.00) | 10.70 (9.00-11.70) | 10.70 (9.60-11.20) | 2.80 (2.70-2.90) | 1351.00 (1261.00-1450.00) | 315.00 (271.00-349.00) | 27.00 (23.00-36.00) |
| *Takydromus wolteri* | 90.50 (32.00-202.50) | 12.70 (11.60-13.60) | 9.70 (8.50-11.03) | 2.70 (2.50-2.90) | 1295.00 (1211.50-1481.00) | 277.50 (252.00-300.00) | 28.00 (24.00-37.00) |
| *Eremias argus* | 22.00 (6.00-60.00) | 12.00 (11.33-12.20) | 9.50 (9.23-11.10) | 2.60 (2.50-2.90) | 1199.00 (1150.25-1273.50) | 289.50 (252.50-324.00) | 26.50 (20-28.75) |
| *Oocatochus rufodorsatus* | 116.50 (47.75-237.00) | 11.50 (10.90-12.40) | 10.90 (9.80-11.40) | 2.80 (2.70-3.00) | 1266.50 (1205.75-1363.00) | 298.00 (256.75-326.00) | 24.00 (22.00-28.00) |
| *Elaphe dione* | 167.00 (61.00-300.00) | 11.60 (10.50-12.80) | 10.60 (9.30-11.20) | 2.80 (2.70-2.90) | 1303.00 (1223.00-1416.00) | 284.00 (252.00-328.00) | 27.00 (23.00-33.00) |
| *Elaphe schrenckii* | 89.00 (16.50-264.50) | 11.70 (10.40-12.55) | 9.70 (8.75-11.20) | 2.70 (2.50-2.90) | 1255.00 (1155.50-1384.00) | 275.00 (259.00-322.50) | 27.00 (22.00-30.50) |
| *Rhabdophis tigrinus* | 150.00 (59.00-276.00) | 11.70 (10.80-12.90) | 10.60 (9.40-11.20) | 2.80 (2.70-2.90) | 1302.00 (1219.00-1423.00) | 285.00 (256.00-320.00) | 26.00 (22.00-32.00) |
| *Hebius vibakari* | 139.00 (49.00-298.00) | 12.90 (10.80-14.70) | 8.90 (7.10-10.60) | 2.70 (2.50-2.80) | 1509.00 (1313.00-1704.00) | 279.00 (262.00-319.00) | 38.00 (25.00-47.00) |
| *Sibynophis chinensis* | 257.00 (183.00-314.00) | 14.10 (13.80-14.70) | 6.70 (6.70-6.80) | 2.40 (2.40-2.40) | 1613.00 (1598.00-1703.00) | 249.00 (246.00-265.00) | 46.00 (46.00-47.00) |
| *Lycodon rufozonatus* | 173.00 (74.50-292.50) | 11.70 (10.80-13.10) | 10.70 (9.30-11.30) | 2.80 (2.70-2.95) | 1313.00 (1230.50-1421.50) | 287.00 (257.00-313.00) | 26.00 (23.00-32.00) |
| *Orientocoluber spinalis* | 29.00 (3.50-124.50) | 13.80 (12.50-14.10) | 8.40 (7.95-10.15) | 2.50 (2.50-2.80) | 1197.00 (1077.00-1315.00) | 237.00 (191.50-263.50) | 32.00 (26.50-33.00) |
| *Gloydius ussuriensis* | 235.00 (116.00-382.00) | 11.20 (10.00-12.20) | 10.70 (9.40-11.30) | 2.80 (2.70-2.90) | 1328.50 (1232.25-1439.75) | 293.50 (254.00-332.75) | 27.00 (23.00-35.00) |
| *Gloydius brevicaudus* | 177.00 (86.00-293.00) | 11.50 (10.60-12.50) | 10.90 (10.00-11.40) | 2.90 (2.70-3.00) | 1282.00 (1215.00-1401.00) | 290.00 (251.00-323.00) | 25.00 (21.00-29.00) |
| *Gloydius intermedius* | 299.50 (163.75-431.50) | 10.40 (9.18-11.50) | 11.00 (10.18-11.43) | 2.80 (2.70-2.90) | 1321.50 (1248.00-1378.25) | 317.50 (275.50-340.25) | 25.00 (22.00-32.00) |

**Supplementary Figure S1**

Species distribution models of the 19 amphibian species present in South Korea. This map was generated using the tool of ArcGIS 10.3 (ESRI, Redlands, CA, USA, [http://www.esri.com](http://www.esri.com/)).

**
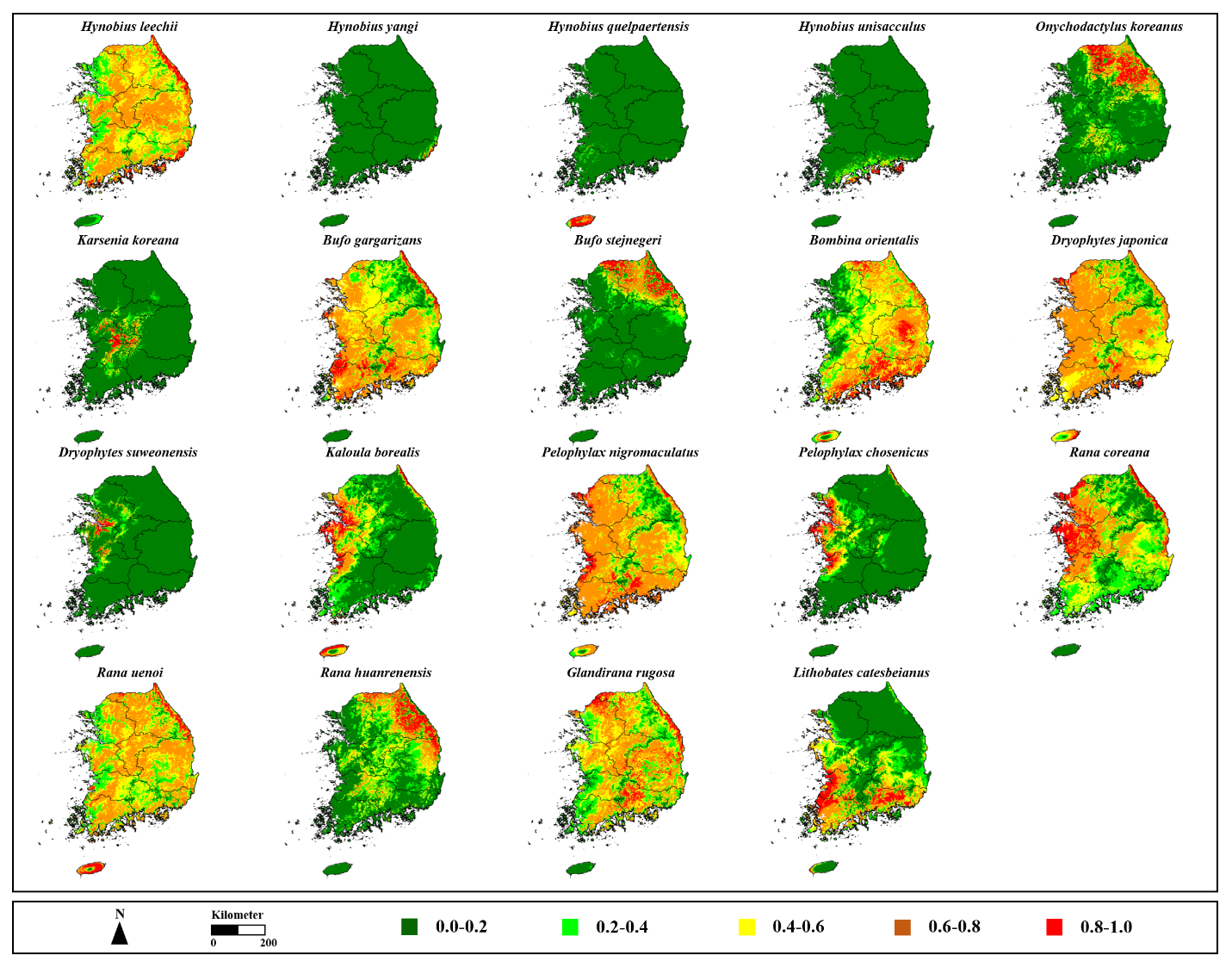
**

**Supplementary Figure S2**

Species distribution models of the 20 reptile species present in South Korea. This map was generated using the tool of ArcGIS 10.3 (ESRI, Redlands, CA, USA, [http://www.esri.com](http://www.esri.com/)).


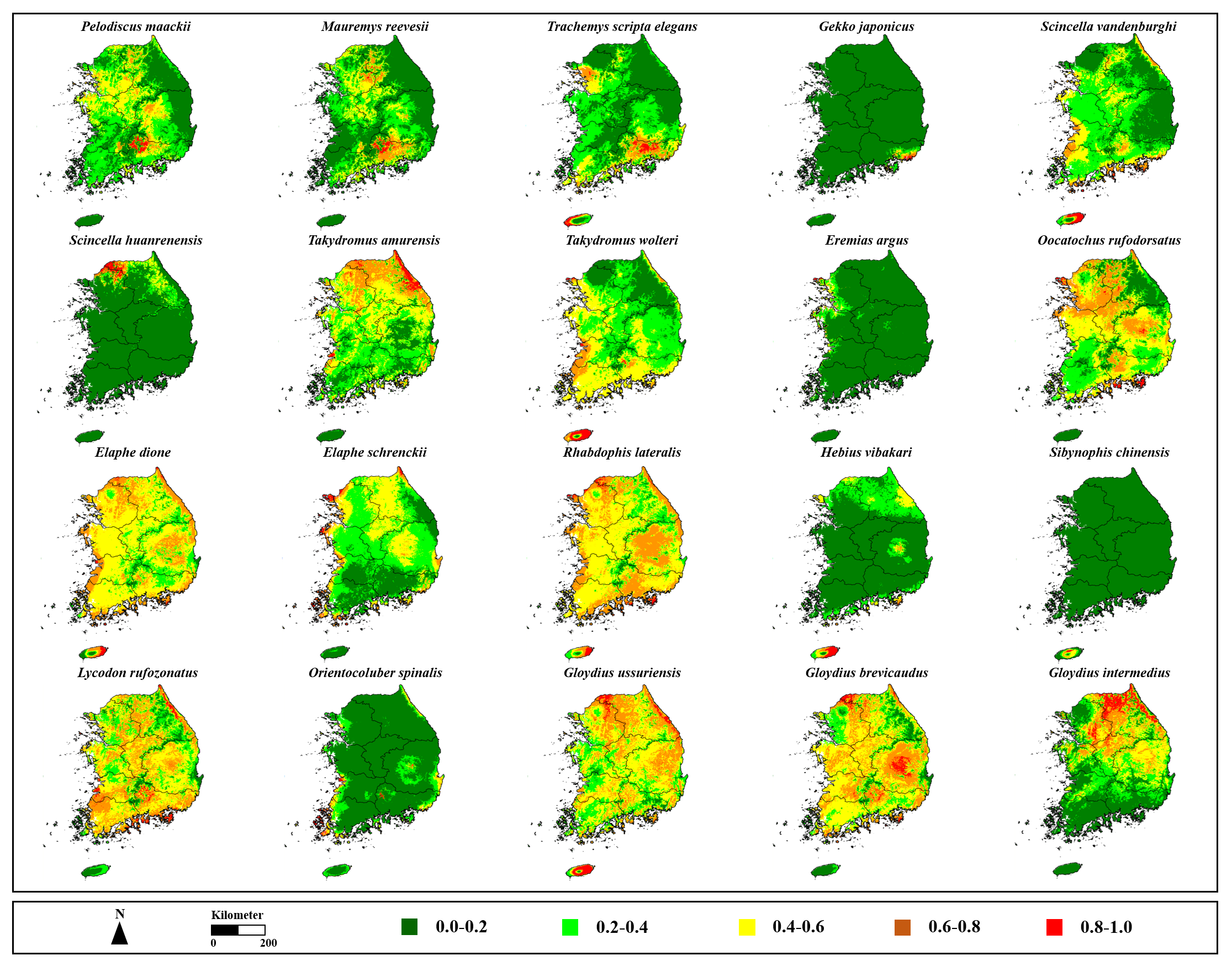

Supplement: Supplementary file 1 — Supplementary Information. [file 41598_2022_19129_MOESM1_ESM.docx]
